# Co-Lifecycle Governance for Learning Medical AI: A Hybrid Convergence Framework for Adaptive Regulatory Oversight

**DOI:** 10.2196/90654

**Published:** 2026-05-19

**Authors:** Jae Hyun Lee, Boram Choi, Kwunho Jeong, Sang Won Suh, Ju Han Kim, Dae-Soon Son

**Affiliations:** 1 Global Research Center, JNPMEDI, Seoul, Republic of Korea; 2 Department of Physiology, College of Medicine, Hallym University, Chuncheon, Gangwon-do, Republic of Korea; 3 Division of Biomedical Informatics, Seoul National University Biomedical Informatics (SNUBI), College of Medicine, Seoul National University, Seoul, Republic of Korea; 4 Major in Bio-Healthcare Convergence, College of Natural Sciences, Hallym University, 1 Hallymdaehak-gil, Chuncheon, Gangwon-do, 24252, Republic of Korea, 82 332482037; 5 Division of Big Data and Artificial Intelligence, Institute of New Frontier Research, College of Medicine, Hallym University, Chuncheon, Gangwon-do, Republic of Korea; 6 Hallym AI-BioHealth R&BD Center, Research Institute of Medical-Bio Convergence, Hallym University, Chuncheon, Gangwon-do, Republic of Korea

**Keywords:** medical artificial intelligence, medical AI, adaptive artificial intelligence, adaptive AI, learning health systems, Co-Lifecycle Governance, regulatory science, United States Food and Drug Administration Predetermined Change Control Plan, European Union AI Act, Digital Medical Products Act, hybrid governance

## Abstract

Artificial intelligence (AI) in health care is increasingly defined not by static algorithms but by adaptive intelligence—systems that evolve over time through interactions with data, clinicians, and clinical environments. This adaptive capacity creates a structural mismatch with regulatory frameworks built for technologies whose behavior remains static. As AI models drift, recalibrate, or degrade in real-world contexts, they dissolve the linear boundaries between design, deployment, and clinical interpretation. These temporal, epistemic, and organizational frictions expose responsibility gaps that cannot be resolved through incremental modifications to legacy oversight structures. Regulators across major jurisdictions are beginning to respond to these challenges, though with differing orientations. The United States advances mechanisms for predictable adaptation, including Predetermined Change Control Plans, real-world evidence frameworks, and life cycle–oriented quality management reforms. The European Union emphasizes precautionary, rights-based governance through the European Union Artificial Intelligence Act (AI Act) and modernized liability rules. South Korea, operating within a hyperconnected digital health ecosystem, has introduced the Digital Medical Products Act (DMPA), one of the world’s first comprehensive statutory frameworks for learning medical AI. Despite philosophical differences, these regulatory trajectories converge on a shared insight: learning AI systems cannot be governed by static rules or episodic evaluation. This viewpoint proposes Co-Lifecycle Governance as a conceptual framework to synchronize regulatory oversight with adaptive intelligence. Rather than treating oversight as a discrete event, Co-Lifecycle Governance frames regulation as a continuous, synchronized process grounded in 4 pillars: continuous validation, agile change management, proactive performance surveillance, and distributed accountability. Each pillar functions as a structural antidote to the responsibility frictions that arise when AI systems evolve faster than expectations surrounding them. Together, these pillars provide a governance grammar capable of supporting safe, iterative model improvement while maintaining system-level trust. Drawing from the strengths of US predictability, European Union accountability, and Korean scalability, this paper outlines a hybrid convergence pathway that synthesizes predictability, accountability, and operational feasibility. Learning AI will not wait for governance to catch up; oversight must evolve in lockstep with adaptive intelligence. Co-Lifecycle Governance offers a foundation for regulatory systems that not only regulate learning AI but also learn with it—at the speed at which adaptive intelligence actually changes.

## Introduction

Medical artificial intelligence (AI) is no longer a fixed device; it is a learning system whose behavior shifts as its data, context, and interactions change. Traditional medical technologies move through segmented and predictable phases, whereas learning AI operates through continuous feedback loops. Evidence from explainability research, clinical deployment challenges, unintended consequences, big data fragility, ethical risks, calibration drift, adversarial vulnerabilities, and explainability limitations demonstrates that learning systems do not maintain a single fixed “performance state” [1-8].

Throughout this viewpoint, we use “learning medical AI” as an umbrella term for AI-enabled medical software whose behavior can change after deployment through planned updates, recalibration, or context-dependent drift. This does not imply autonomous real-time online learning at the bedside, which is neither assumed nor advocated in this framework. In many medical domains, definitive outcome labels are delayed (“ground truth lag”), which makes life cycle governance dependent on staged reassessment rather than continuous real-time outcome learning. Accordingly, throughout this viewpoint, we use “continuous” strictly in a life cycle sense—ongoing, repeated, and trigger-based—rather than as uninterrupted real-time outcome learning.

This paper begins from a consequential insight: the life cycle of a medical product and the learning cycle of an AI system are structurally distinct yet increasingly intertwined. Embedding AI into clinical environments reshapes its behavior in ways that static regulatory snapshots cannot adequately represent. As a result, foundational governance questions arise: Who is responsible when models drift? How should updates be reviewed? Which evidence should guide continuous performance assessment?

Regulators worldwide are experimenting with different answers. The United States is developing structured pathways for predictable adaptation, such as Predetermined Change Control Plans (PCCPs) [9-11]. The European Union (EU) emphasizes precautionary accountability through the AI Act and accompanying liability reforms [12,13]. Korea has enacted the DMPA, establishing a statutory foundation for adaptive oversight within a highly digitized health care ecosystem [14].

Taken together, these regulatory responses point to a shared structural realization: learning medical AI cannot be effectively governed through static rules or episodic review. While each jurisdiction approaches the problem from a distinct regulatory philosophy—adaptive flexibility in the United States, precautionary accountability in the EU, and infrastructural hybridization in Korea—all 3 are converging on the need for oversight systems that remain responsive to ongoing model evolution. These systems share the same problem statement, but they operationalize it through different regulatory logics. This convergence, coupled with persistent divergence in implementation, creates the conditions for a governance framework that can integrate adaptability, accountability, and operational feasibility within a single life cycle–aware structure.

This viewpoint proposes Co-Lifecycle Governance, a framework that integrates regulatory, organizational, and technical responsibilities to synchronize oversight with adaptive intelligence. Rather than replicating any single jurisdictional model, Co-Lifecycle Governance articulates a life cycle–aware structure capable of operationalizing the core requirements embedded across PCCP-based update governance, risk-based AI regulation, and statutory digital medical product oversight.

## Regulatory DNA of Learning AI: Divergent Philosophies and Converging Pressures

Regulatory systems designed for fixed-performance technologies were never built to follow AI that continues to change itself after approval. What distinguishes learning AI is not only its evolving performance but also the mechanisms through which that evolution emerges—data drift, contextual adaptation, workflow coupling, and distributed system interactions. These dynamics force regulators to articulate what kinds of change should be permitted, anticipated, monitored, or constrained. The result has been the crystallization of 3 regulatory archetypes, each representing a deeper institutional philosophy about uncertainty, risk, and technological evolution. Although they often appear as regional policy variations, they are better understood as expressions of distinct regulatory “genotypes”: different ways of encoding how a governance system adapts to the presence of learning systems.

In this context, “regulatory DNA”—previously articulated in comparative form between the United States and the EU [15]—does not refer to a normative ideal or a shared philosophical blueprint. Rather, it describes the structural response patterns that different regulatory systems have activated in reaction to the same underlying pressure: the inability of fixed, static regulatory models to govern learning systems that continue to change after deployment. While the United States, the EU, and Korea exhibit divergent regulatory forms, they are responding to a common constraint—the need to govern AI across its life cycle rather than at a single point in time.

## United States: Predictable Adaptation Through Structured Flexibility

The United States has taken the clearest steps toward treating model evolution not as a postmarket anomaly but as a governable life cycle property. The United States Food and Drug Administration (FDA)’s introduction of PCCPs [9] represents a foundational shift in regulatory logic: manufacturers may prespecify which aspects of a model will change, how they will change, and what validation evidence will be required before updates are deployed. This preauthorization mechanism acknowledges that learning systems cannot be frozen at the moment of approval. Instead, it reframes updates as predictable events within a predefined envelope of acceptable change.

This philosophy has roots in the FDA’s long-standing Total Product Life Cycle approach, which emphasizes that the safety of software-based products is shaped not only by design but also by postmarket performance and the manufacturer’s quality processes. The modernization of the Quality Management System Regulation [11] reinforces this orientation by embedding continuous monitoring, process discipline, and organizational accountability into regulatory expectations. Together with the International Medical Device Regulators Forum’s foundational software as a medical device (SaMD) frameworks (definitions, risk categorization, and clinical evaluation) [16-18], these instruments operationalize a life cycle–thinking mindset in which regulators oversee systems in motion, not artifacts at rest.

Importantly, the US governance approach also relies on real-world evidence (RWE) to monitor performance and validate changes [10]. RWE offers regulators a dynamic evidentiary substrate for detecting drift, inequities, or clinically significant shifts that may not appear during premarket testing. This integration of real-world learning into regulatory oversight represents a pragmatic recognition: learning AI is safer when regulators explicitly permit controlled evolution rather than forcing manufacturers to choose between compliance and improvement.

Still, the US approach carries trade-offs. Its emphasis on developer responsibility and on organizational quality systems places substantial trust in manufacturers to monitor themselves. Moreover, while PCCPs offer clarity about planned updates, they provide less formal structure for unplanned evolution arising from distributional drift or environmental coupling—precisely the kinds of changes most likely to generate risk. Nevertheless, as an archetype, the US model contributes a vital regulatory virtue: predictable adaptation, which aligns closely with the Co-Lifecycle Governance pillar of agile change management.

## EU: Precautionary Governance Anchored in Accountability

The EU approaches learning AI through a fundamentally different regulatory lens. Where the United States structures change, the EU seeks to control risk and ensure rights. The AI Act [12] is built on a precautionary logic: high-risk AI systems—especially those used in medical contexts—must undergo extensive ex ante conformity assessment, maintain comprehensive technical documentation, demonstrate risk mitigation measures, and preserve human oversight throughout their life cycle. The premise is not that AI will necessarily evolve safely, but that institutions must be able to reconstruct and audit system behavior at every stage.

This approach reflects a long-standing European regulatory tradition: documentation as governance. By requiring detailed data provenance records, model design dossiers, transparency reports, and continuous postmarket monitoring plans, the AI Act constructs an auditable trail that can reveal how a model changes, how those changes were validated, and how they may contribute to harm. In this sense, the EU does not primarily regulate learning as a dynamic engineering process; rather, it regulates the evidence environment needed to hold systems accountable.

The updated Product Liability Directive (PLD) [13] further reinforces this architecture by expanding liability to software-driven harms and update-related failures. Under the new PLD, manufacturers may face strict liability if insufficient documentation prevents courts from determining how a failure occurred. This makes transparency not only a compliance requirement but also a legal survival mechanism. If a model’s learning trajectory is not well documented, its developer becomes more—not less—exposed.

Yet the EU approach has its own trade-offs. Its strong ex ante compliance burden can slow down adaptation, creating tension for AI systems that rely on frequent updates to remain safe or clinically relevant. The AI Act permits modifications, but the pathways for high-risk systems remain comparatively rigid, requiring additional documentation and potential reassessment. At scale, this may create a mismatch between the speed of learning and the velocity of regulatory processes.

Still, the EU model contributes a distinct regulatory strength that no other jurisdiction provides as strongly: rights-based accountability. It articulates a governance stance in which AI evolution is allowed only when it remains transparent, contestable, and traceable. In Co-Lifecycle Governance terms, the EU strengthens the pillar of distributed accountability, ensuring that oversight does not simply trust manufacturers but constrains them through enforceable obligations.

## Korea: Hybridization in a Hyperconnected Ecosystem

South Korea offers a regulatory trajectory shaped not only by policy choice but also by structural conditions that make fixed, static oversight particularly difficult to sustain. As one of the world’s most digitally connected health care environments—with near-universal electronic health record adoption, integrated claims databases, nationwide broadband infrastructure, and high digital literacy—Korea operates in a setting where AI-enabled medical products are likely to evolve rapidly once deployed. In such an environment, regulatory frameworks that rely solely on premarket authorization or episodic review are inherently mismatched to real-world use. The DMPA [14,19] emerges from this context as one of the first statutory frameworks explicitly designed to govern digital medical technologies, including adaptive AI, across their operational life cycle.

In this sense, the DMPA does not simply borrow from US or European regulatory traditions; it reflects a convergence driven by necessity. Korea’s regulatory environment faced simultaneous pressures: the need to accommodate iterative software updates without stalling clinical innovation, and the need to ensure traceability, documentation, and enforceability in a highly data-intensive health system. Elements commonly associated with US governance—such as acceptance of planned postmarket modification and structured update pathways—are reflected in the Ministry of Food and Drug Safety (MFDS) guidance emphasizing proportionality and predefined validation of changes [20]. At the same time, the DMPA incorporates features characteristic of European regulation, including explicit documentation requirements and auditable risk management structures, which are essential in a system where large-scale data integration amplifies both benefit and harm.

Korea’s broader Digital Health Innovation Strategy [21] further reinforces this hybridization by embedding AI governance within national health care modernization. Institutional coherence across the MFDS, the Health Insurance Review and Assessment Service, the National Institute of Medical Device Safety Information, and clinical providers creates conditions under which postmarket oversight can be coordinated at scale. Importantly, this infrastructural strength should not be interpreted as implying continuous raw-data access for private developers to retrain models. Rather, Korea’s centralized claims and safety-monitoring systems primarily enable mediated, privacy-preserving surveillance and audit mechanisms. Under such an approach, population-level data can support regulator- or institution-led monitoring, signal detection, and periodic revalidation without necessitating unrestricted data transfer to manufacturers. In this sense, the infrastructure strengthens the learning cycle of oversight and recalibration. It does not imply autonomous, developer-driven continuous model updating. At the same time, centralized oversight architectures introduce their own governance trade-offs, including heightened privacy sensitivity, potential centralization risks, and institutional dependency. These constraints should be recognized as part of the design conditions for scalable life cycle governance, not as automatic advantages. The boundary between medical AI and broader health care AI is particularly salient in Korea’s fast-moving digital ecosystem. Tools that begin as wellness, administrative, or population-health software (eg, lifestyle coaching, symptom checkers, appointment triage, or operational risk stratification) may become “medical” AI once their intended use shifts toward diagnosis, treatment selection, or clinical risk management. For learning systems, this boundary can evolve over time as workflow integration deepens, creating “category drift,” which challenges static regulatory categorization and reinforces the need for life cycle–aware governance.

A practical Korean example of boundary management can be seen in the MFDS’s 2020 approval of Samsung Electronics’ blood pressure measurement mobile app as a SaMD [22]. Rather than treating the broader mobile platform as a medical device in its entirety, the MFDS attached device oversight to the specific software function and its intended use: once the app claimed noninvasive measurement and display of blood pressure and pulse, it became subject to medical device performance standards and approval [22]. Although this case is not itself a postdeployment learning event, it demonstrates the operative Korean rule that regulatory status follows function and intended use rather than the digital platform label alone. For adaptive AI, the same logic implies that category drift can occur when iterative updates or deeper workflow integration transform a health-management tool into clinically actionable measurement, triage, or treatment-support software.

These structural conditions directly shape how the 4 pillars of Co-Lifecycle Governance are operationalized in Korea. Surveillance and agile change management are most visibly enabled by Korea’s infrastructure, but the remaining pillars are equally implicated. Continuous validation becomes practicable because performance can be reassessed using real-world population data rather than isolated device-level follow-up studies. Distributed accountability is reinforced by the ability to trace responsibility across developers, deploying institutions, and regulators through unified data and documentation pathways. Rather than emphasizing a single pillar, Korea’s contribution lies in demonstrating how continuous validation, change management, surveillance, and accountability can operate simultaneously within one statutory and infrastructural ecosystem.

The DMPA is still evolving, and implementation challenges remain. Yet Korea’s approach illustrates a form of operational feasibility that neither US flexibility nor European accountability alone can guarantee. Where the US model excels at structuring change and the EU model excels at enforcing responsibility, Korea shows how life cycle–aware governance can be instantiated at scale. In this respect, Korea’s experience is not merely nationally specific but offers a transferable reference for health systems seeking to align regulatory oversight with the realities of learning AI in data-rich environments.

## Convergence

Despite philosophical divergence, the regulatory genotypes expressed in the US, EU, and Korean frameworks converge on a shared structural recognition: learning AI cannot be governed through fixed rules designed for static technologies. Whether through adaptation envelopes, rights-based documentation regimes, or infrastructural hybridization, each jurisdiction is articulating mechanisms for continuous oversight, proportional risk management, and distributed responsibility. Beyond national regulation, international bodies have similarly emphasized the need for continuous governance, responsible data stewardship, and life cycle–aware oversight in digital health and AI systems [23,24]. These 3 regulatory archetypes are summarized in Table 1.

**Table 1.** Comparative regulatory DNA of the United States, European Union, and Korea.

	United States	European Union	Korea
Philosophy	Predictable adaptation	Precautionary accountability	Hybrid operationalization
Key regulatory instruments	PCCP^a^, RWE^b^, and QMSR^c^	AI Act^d^ and PLD^e^	DMPA^f^
Oversight mode	Preauthorized change	High-risk controls	Mixed adaptive pathways
Liability	Tort^g^ + regulatory	Strict liability	Hybrid evolving
Strength	Predictability	Accountability	Scalability

a PCCP: Predetermined Change Control Plan.

b RWE: real-world evidence.

c QMSR: Quality Management System Regulation.

d AI Act: European Union Artificial Intelligence Act.

e PLD: Product Liability Directive.

f DMPA: Digital Medical Products Act.

g Tort refers to civil liability for wrongful acts or omissions, including negligence or product-related harm, outside contractual obligations.

## The Erosion of Linear Responsibility: Structural Frictions in Learning AI

As AI systems evolve during real-world use, responsibility across developers, clinicians, institutions, and regulators becomes unstable. Drift, opacity, and adversarial vulnerabilities erode the linear chain linking design to deployment [1,4-8]. Responsibility gaps arise from three structural frictions:

1. A temporal friction, in which performance changes after approval in ways not reflected in initial validation [6]
2. An epistemic friction, in which failures become invisible to clinicians due to opacity and design constraints [1,7,8]
3. An organizational friction, driven by distributed control across developers, vendors, institutions, and regulators [25-28]

These frictions produce responsibility gaps and “accountability overload,” in which responsibility is simultaneously diluted across multiple actors and disproportionately concentrated on those least able to influence system behavior. Learning AI thus destabilizes legacy responsibility doctrines that rely on static role allocation and linear chains of control. Addressing this breakdown requires a governance structure capable of tracking responsibility across time, reallocating obligations as systems evolve, and maintaining traceability between technological change and regulatory accountability. The resulting life cycle responsibility matrix is summarized in Table 2.

**Table 2.** Responsibility matrix across the life cycle of learning artificial intelligence systems.

	Developer	Institution	Clinician	Regulator
Design	Architecture	—^a^	—	Standards
Deployment	Documentation	Integration quality	Correct use	Authorization
Real-world learning	Patch cycles	Monitoring	Interpretation	Surveillance
Failure	Root cause	Incident detection	Reporting	Enforcement
Update	Validation	Rollout oversight	Communication	PCCP^b^ and DMPA^c^ rules

a No primary responsibility is assigned to that actor at the corresponding life cycle stage.

b PCCP: Predetermined Change Control Plan.

c DMPA: Digital Medical Products Act.

These structural frictions point to a deeper limitation of existing governance approaches. Learning medical AI systems do not merely introduce new risks; they alter the temporal and organizational conditions under which responsibility is exercised. Addressing these challenges therefore requires a governance framework that treats technological change as a core design feature rather than an exception to be managed after deployment. The central task is not only to allocate responsibility when failures occur but also to synchronize regulatory oversight with the ongoing evolution of learning systems.

## Co-Lifecycle Governance: 4 Pillars for Learning Systems

**Figure 1.**
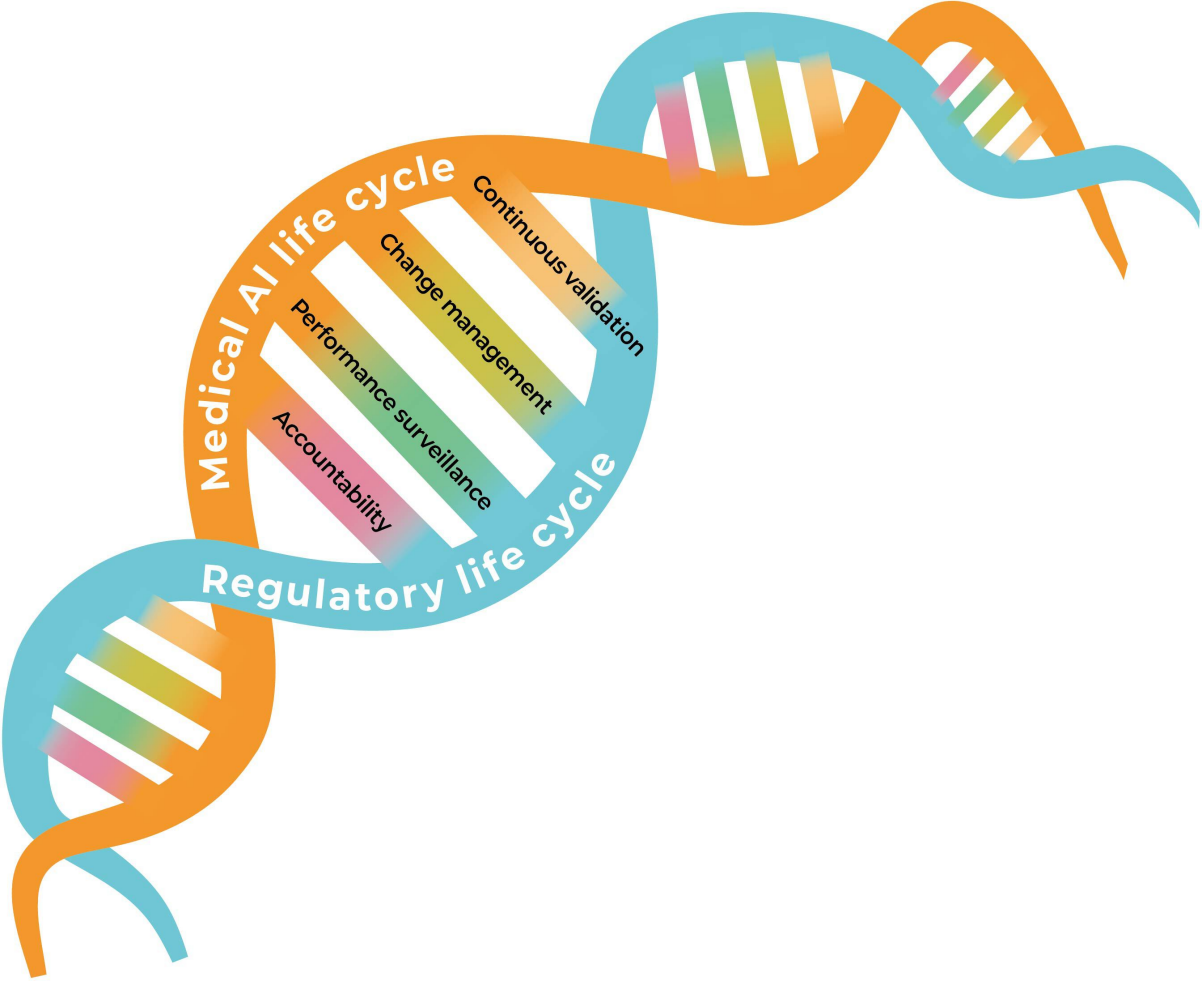
Co-Lifecycle Governance framework for learning medical artificial intelligence (AI). This figure illustrates the parallel and interconnected evolution of medical AI systems and regulatory oversight across the product life cycle.

Co-Lifecycle Governance operationalizes the coevolutionary relationship between learning medical AI and regulatory oversight through 4 interdependent pillars. Rather than functioning as isolated controls, these pillars work together to ensure that adaptation remains both governable and accountable across the product life cycle, as illustrated in Figure 1.

The first pillar, continuous validation, addresses temporal instability introduced by learning systems. Because performance and calibration may shift as data distributions and clinical workflows evolve, validation cannot remain confined to premarket evaluation. However, “continuous” validation is not synonymous with real-time outcome learning. In settings with delayed end points (eg, oncology or chronic disease), validation must operate across time horizons: (1) short-horizon monitoring of input drift, uncertainty, and process indicators; (2) intermediate validation using proxy outcomes, chart-review audits, or surrogate markers; and (3) longer-horizon confirmation once definitive outcomes mature. This staged structure allows governance to respond to drift early while preserving scientific rigor when ground truth is lagged [2,4,6]. Continuous validation reframes regulatory assurance as an ongoing life cycle obligation, not a one-time premarket certification event. Such life cycle–based oversight inevitably entails financial, technical, and organizational burdens, which must be proportionately allocated across developers, institutions, and regulators. In practice, these burdens may be addressed through structured cost-sharing mechanisms. For example, multi-institutional data-sharing consortia can distribute validation costs across participating sites, while subscription-based service models may incorporate ongoing surveillance and validation as part of software maintenance. In some settings, public infrastructure may support baseline monitoring functions, reducing the burden on individual institutions.

The second pillar, agile change management, structures how updates are proposed, validated, authorized, communicated, and rolled out. Mechanisms such as PCCPs and risk-proportionate pathways under the DMPA exemplify how model evolution can be made transparent, reviewable, and predictable [9,14]. Importantly, agility must be paired with version governance to avoid inequities across care environments. Co-Lifecycle Governance therefore treats version transparency (who is running which model, with what validated performance), minimum performance floors, and update dissemination policies as ethical and operational requirements rather than mere engineering choices.

The third pillar, proactive performance surveillance, responds to the epistemic opacity of learning AI by providing early warning signals. Surveillance is primarily passive and signal-oriented: it continuously tracks aggregate and subgroup performance proxies (eg, drift metrics, calibration signals, alert rates, and incident reports) to identify anomalies that warrant investigation [1,7,8]. By design, surveillance does not “reprove” effectiveness; it detects deviations and triggers targeted validation when thresholds are crossed. This operational distinction clarifies why surveillance and validation can share data infrastructures yet remain functionally nonredundant.

Finally, distributed accountability aligns obligations with actual control across developers, deploying institutions, clinicians, and regulators. Here, “distributed” refers to operational responsibility (who monitors, investigates, communicates, and executes mitigations), not the dilution of legal liability. In strict liability regimes, financial liability may remain concentrated on manufacturers, but operational accountability must still be traceable across the sociotechnical system. Co-Lifecycle Governance therefore emphasizes traceability: a chain of custody for model versions, validation evidence, deployment context, and corrective actions, which supports both clinical trust and legal enforceability [25-28]. This distinction preserves compatibility with strict liability regimes, where enforceability depends on clear forensic attribution. This structure also remains compatible with the learned intermediary doctrine, under which clinicians retain final decision-making authority in patient care.

For this structure to remain clinically viable, explainability must function as a deployment and update authorization requirement rather than an optional design feature. Initial deployment and any material recalibration or postmarket update must be conditioned on evidence that the system provides interpretable outputs, uncertainty signals, and traceable decision pathways sufficient for clinicians to understand the basis, confidence, and limits of model recommendations within the relevant workflow. Continuous validation must reassess these explainability properties after substantial model or interface changes, and performance surveillance should treat systematic clinician confusion, abnormal override patterns, or unexplained subgroup performance shifts as safety signals. Where these conditions cannot be met, the system should not be deployed or should be restricted to narrower use contexts, because clinician authority would otherwise be reduced to nominal endorsement rather than meaningful mediation. In this sense, explainability is not merely desirable; it is a governance gate that preserves both clinical trust and the practical viability of the learned intermediary doctrine.

Taken together, these 4 pillars provide coordinated answers to the structural frictions identified earlier: continuous validation mitigates temporal drift, performance surveillance addresses epistemic opacity, and distributed accountability—working in concert with agile change management—resolves organizational fragmentation. In combination, they form a life cycle–aware governance architecture capable of synchronizing technological evolution with regulatory expectations.

A robust life cycle governance framework must also specify an exit strategy. When surveillance and validation indicate persistent, nonmitigable risk, governance should support staged controls: conditional suspension (use restricted to defined contexts), rollback to a prior validated version, and decommissioning or recall when risks cannot be acceptably reduced. We position this “safe exit” protocol as a necessary complement to update pathways, ensuring that life cycle governance is not biased toward improvement alone.

However, executing rollback or decommissioning in clinical environments is not frictionless. Technical dependencies between AI systems and electronic health record infrastructures may complicate rapid reversion to prior versions. Abrupt changes in system behavior may also disrupt clinical workflows and introduce new safety risks, particularly if clinicians have adapted to the updated system. These constraints highlight the need for preplanned and institutionally integrated exit strategies. Table 3 maps the 4 pillars of Co-Lifecycle Governance to representative regulatory anchors in the United States, EU, and Korea.

**Table 3.** Mapping the 4 pillars of Co-Lifecycle Governance to regulatory anchors.

Pillar	United States	European Union	Korea
Continuous validation	RWE^a^ and QMSR^b^	AI Act^c^ documentation	DMPA^d^ performance tracking
Agile change management	PCCP^e^	Change subject to conformity assessment and recertification triggers	Adaptive update pathways
Surveillance	RWE	Mandatory monitoring	National-scale infrastructure
Accountability	Shared	Strict liability	Hybrid

a RWE: real-world evidence.

b QMSR: Quality Management System Regulation.

c AI Act: European Union Artificial Intelligence Act.

d DMPA: Digital Medical Products Act.

e PCCP: Predetermined Change Control Plan.

Because continuous validation and performance surveillance both rely on postmarket data streams, Table 4 clarifies their distinct operational roles.

**Table 4.** Operational comparison between continuous validation and performance surveillance.

Dimension	Continuous validation (pillar 1)	Surveillance (pillar 3)
Primary purpose	Confirm or update performance claims	Detect early warning signals
Mode	Active, hypothesis-driven testing	Passive, continuous monitoring
Evidence standard	Higher (reference standards and audits)	Preliminary (proxies and alerts)
Typical data	Curated labeled sets, chart review, surrogate outcomes, and matured end points	Drift metrics, calibration signals, alert rates, incident reports, and subgroup signals
Trigger	Scheduled revalidation or triggered by surveillance	Always on and triggers validation when thresholds are crossed
Output	Revalidation report, updated performance claims, and recalibration decision	Alerts/flags, monitoring reports, and escalation tickets
Decision action	Approve recalibration or update or safe exit	Escalate to validation and change control

## Illustrative Clinical Scenario

To illustrate how the proposed framework operates in practice, consider a sepsis prediction algorithm deployed in an intensive care unit.

Over time, clinicians begin to observe fewer alerts for high-risk patients, potentially increasing the risk of delayed intervention in deteriorating patients. Concurrently, performance surveillance systems detect a decline in calibration across specific patient subgroups (pillar 3), triggering an institutional alert.

This signal initiates targeted validation (pillar 1), in which recent patient data and chart review audits are used to assess whether the observed drift reflects true performance degradation. Upon confirmation, the developer implements a recalibration update under a prespecified change management plan (pillar 2), with updated performance metrics communicated to clinicians prior to deployment.

Throughout this process, version tracking and audit logs ensure traceability of decisions and responsibility allocation (pillar 4). If recalibration fails to restore acceptable performance, the system may enter a staged exit pathway, including conditional suspension or rollback.

## Hybrid Convergence: Synthesizing Predictability, Accountability, and Scale

As previously analyzed in comparative context [15], the United States contributes predictable adaptation through PCCPs, RWE, and life cycle–oriented quality systems [9-11,16-18]. The EU contributes enforceable accountability through risk-based governance and liability reform under the AI Act and PLD [12,13]. Korea contributes scalable operationalization through statutory digital medical product frameworks and nationally coordinated digital health infrastructure [14,19-21].

**Figure 2.**
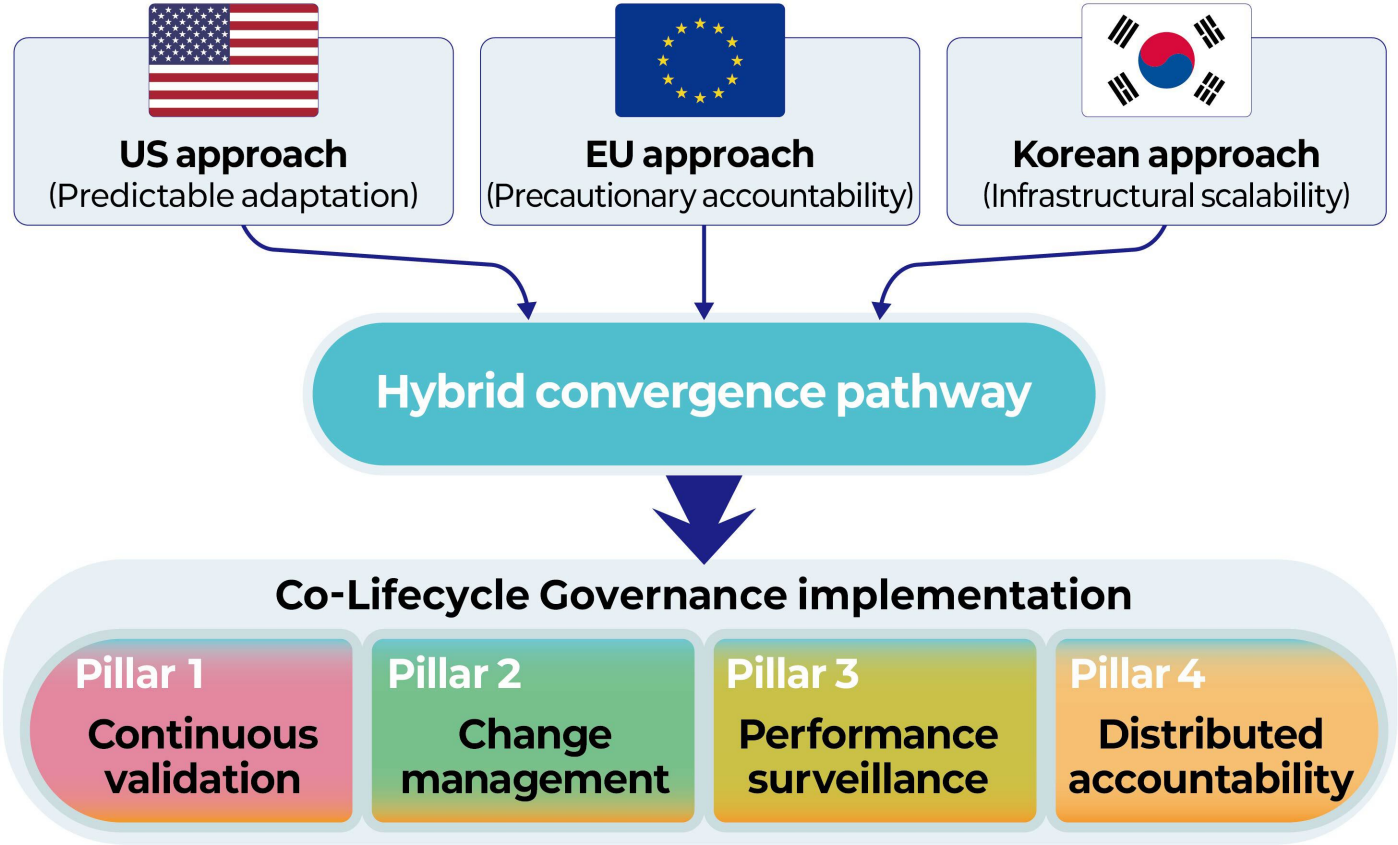
Convergence of archetypal regulatory logics into a hybrid life cycle governance architecture. This schematic figure shows how the United States (predictability), the European Union (EU; accountability), and Korea (scalability) converge toward a shared hybrid life cycle governance architecture.

Rather than proposing a single harmonized regulatory procedure, the hybrid convergence should be understood as a governance grammar—a set of life cycle concepts and operational primitives that can be instantiated within different legal traditions. In practice, manufacturers operate under one primary jurisdiction at a time, while global developers may translate shared governance artifacts (eg, version traceability records, validation documentation, and monitoring plans) into jurisdiction-specific compliance packages. Figure 2 illustrates this convergence pathway: distinct regulatory logics feed into a shared life cycle–oriented governance architecture without requiring legal harmonization.

This framing addresses the apparent friction between rapid iteration and precautionary compliance. US-style preauthorized change pathways enable structured model updates, whereas the EU high-risk regime emphasizes conformity assessment, documentation, and recertification triggers. Co-Lifecycle Governance does not claim to eliminate these compliance burdens. Instead, it renders them governable by (1) classifying changes according to risk and regulatory impact, (2) prespecifying validation evidence proportional to each change category, and (3) maintaining audit-ready documentation that preserves traceability across updates. In this sense, agile change management can operate within precautionary systems as a discipline for anticipating and controlling recertification thresholds rather than as a mechanism for unconstrained speed.

The focus on the United States, EU, and Korea reflects their representation of 3 archetypal regulatory logics—predictable adaptation, precautionary accountability, and infrastructural scalability—rather than an exhaustive global survey.

Other jurisdictions, including Japan’s postapproval change management approaches, Singapore’s life cycle guidance for software medical devices, and China’s classification principles for AI-based medical software, demonstrate parallel movement toward life cycle–aware governance. China has also issued classification and technical review guidance for AI-based medical software, emphasizing risk-based categorization and performance evaluation requirements tied to intended use and clinical function. Japan has explored controlled postapproval update pathways, while Singapore emphasizes life cycle monitoring and quality system–based regulatory flexibility. These examples reinforce the broader claim of structural convergence while preserving regional divergence in implementation.

Finally, scalability is not synonymous with simplicity. National infrastructures that enable coordinated surveillance also introduce governance prerequisites and risks, including robust data governance, privacy safeguards, institutional capacity for monitoring, and clear legal pathways for corrective action. Co-Lifecycle Governance treats these as design constraints to be explicitly managed rather than as automatic advantages. Therefore, the hybrid convergence represents not a unified regime but a convergent architecture through which diverse regulatory systems can align life cycle oversight with adaptive intelligence.

In the context of unplanned postmarket drift, regulatory approaches also diverge. In the United States, such events are primarily addressed through manufacturer-led monitoring within quality management systems, with less formalized pathways for unplanned changes outside PCCP-defined updates. In contrast, the Korean framework enables more centralized signal detection through national-scale infrastructure, supporting coordinated revalidation and corrective action. This distinction reflects a broader difference between distributed monitoring and infrastructure-enabled oversight.

## Conclusions

Learning AI exposes foundational limits of legacy governance structures. Evidence of drift, opacity, and adversarial vulnerability demonstrates why static oversight cannot ensure safety in dynamic environments [1,4-8]. Co-Lifecycle Governance reframes oversight as a synchronized, adaptive process grounded in 4 structural pillars [2-8,25-28]. These pillars provide a shared language for distributing responsibility and coordinating obligations across the life cycle.

Internationally, predictable adaptation (United States) [9-11], enforceable accountability (EU) [12,13], and operational scalability (Korea) [14,19-21] represent complementary strengths. No single philosophy suffices in isolation. A durable hybrid governance pathway must synthesize these approaches while maintaining public trust.

The pace of adaptive AI development increasingly challenges the capacity of static governance models to respond. Co-Lifecycle Governance offers a foundation for regulatory systems capable of learning and adapting with the technologies they oversee.

## Abbreviations

AI: artificial intelligence
AI Act: European Union Artificial Intelligence Act
DMPA: Digital Medical Products Act
EU: European Union
FDA: United States Food and Drug Administration
MFDS: Ministry of Food and Drug Safety
PCCP: Predetermined Change Control Plan
PLD: Product Liability Directive
RWE: real-world evidence
SaMD: software as a medical device
